# Redetermination of (acetonitrile-κ*N*)dicarbon­yl(η^5^-cyclo­penta­dien­yl)iron(II) tetra­fluoridoborate

**DOI:** 10.1107/S1600536812027857

**Published:** 2012-06-23

**Authors:** Theresa Kückmann, Hans-Wolfram Lerner, Michael Bolte

**Affiliations:** a Institut für Anorganische Chemie, J. W. Goethe-Universität Frankfurt, Max-von-Laue-Strasse 7, 60438 Frankfurt/Main, Germany

## Abstract

The crystal structure of the title compound, [Fe(C_5_H_5_)(CH_3_CN)(CO)_2_]BF_4_, of which only the coordinates of the non-H atoms of the cation have previously been reported [Fadel *et al.* (1979 ▶). *Z. Anorg. Allg. Chem.* **453**, 98–106] has been redetermined. The Fe^II^ atom in the complex cation is coordinated by a cyclo­penta­dienyl ring, two carbonyl ligands and an acetonitrile mol­ecule displaying a three-legged piano stool structure. Three of the four F atoms of the BF_4_^−^ anion are disordered over two sets of sites, with a site-occupancy factor of 0.709 (10) for the major occupied site.

## Related literature

For background to this work, see: Kückmann *et al.* (2005 ▶, 2007 ▶, 2008 ▶, 2010 ▶); Lerner (2005 ▶); Sänger *et al.* (2012 ▶). For a previous (incomplete) structure determination of the title compound, see: Fadel *et al.* (1979 ▶). For the structure of closely related dicarbonyl-(η^5^-cyclo­penta­dien­yl)-(*N*-methyl cyanido)iron(II) tetra­fluoridoborate, see: Callan *et al.* (1987 ▶).
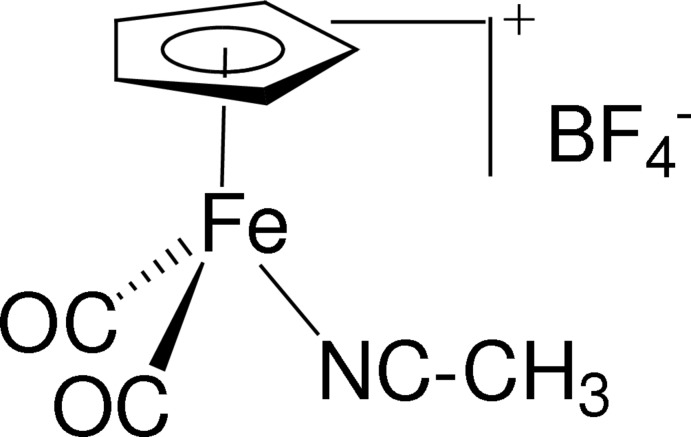


## Experimental

### 

#### Crystal data

[Fe(C_5_H_5_)(C_2_H_3_N)(CO)_2_]BF_4_
*M* *_r_* = 304.82
Monoclinic, 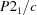

*a* = 6.8842 (7) Å
*b* = 15.289 (2) Å
*c* = 11.4353 (12) Å
β = 95.192 (8)°
*V* = 1198.7 (2) Å^3^
*Z* = 4
Mo *K*α radiation
μ = 1.30 mm^−1^
*T* = 173 K
0.33 × 0.12 × 0.12 mm

#### Data collection

Stoe IPDS II two-circle diffractometer
Absorption correction: multi-scan (*MULABS*; Spek, 2009 ▶; Blessing, 1995 ▶) *T*_min_ = 0.674, *T*_max_ = 0.860
6372 measured reflections
2222 independent reflections
1976 reflections with *I* > 2σ(*I*)
*R*_int_ = 0.044

#### Refinement

*R*[*F* ^2^ > 2σ(*F* ^2^)] = 0.055
*wR*(*F* ^2^) = 0.151
*S* = 1.04
2222 reflections
162 parameters
45 restraints
H-atom parameters constrained
Δρ_max_ = 1.43 e Å^−3^
Δρ_min_ = −0.87 e Å^−3^

### 

Data collection: *X-AREA* (Stoe & Cie, 2001 ▶); cell refinement: *X-AREA*; data reduction: *X-AREA*; program(s) used to solve structure: *SHELXS97* (Sheldrick, 2008 ▶); program(s) used to refine structure: *SHELXL97* (Sheldrick, 2008 ▶); molecular graphics: *XP* in *SHELXTL* (Sheldrick, 2008 ▶); software used to prepare material for publication: *SHELXL97*.

## Supplementary Material

Crystal structure: contains datablock(s) I, global. DOI: 10.1107/S1600536812027857/wm2643sup1.cif

Structure factors: contains datablock(s) I. DOI: 10.1107/S1600536812027857/wm2643Isup2.hkl

Additional supplementary materials:  crystallographic information; 3D view; checkCIF report

## Figures and Tables

**Fig. 1.**
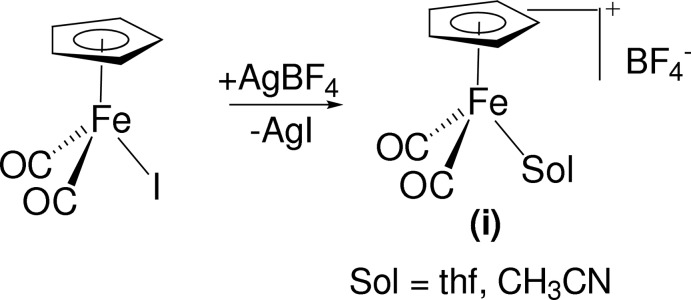
Reaction scheme for obtaining the title compound.

**Fig. 2.**
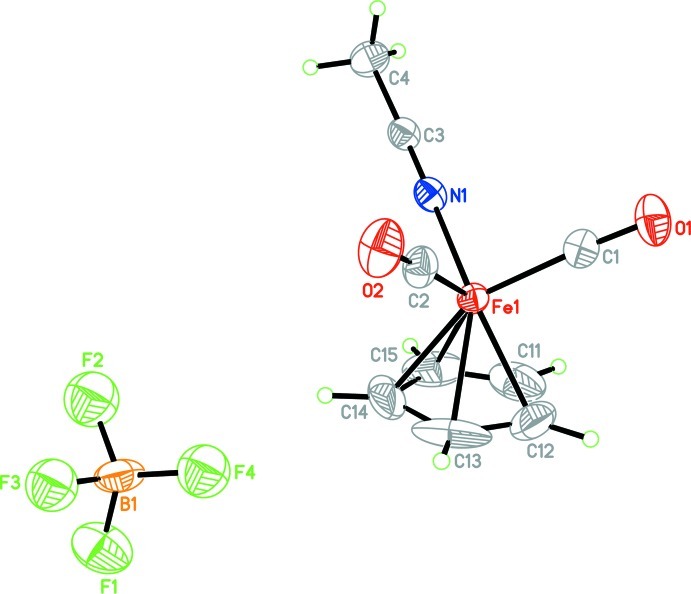
Perspective view of the title compound with the atom numbering scheme; displacement ellipsoids are at the 50% probability level. Only the major occupied site of the disordered BF4- anion is shown.

## supplementary crystallographic information

### Comment

Very recently we have reported (Sänger *et al.*, 2012) that the dimeric iron carbonyl [CpFe(CO)_2_]_2_ and the iodosilanes *t*Bu_2_RSiI (*R* = Me, *t*Bu) were obtained from the reaction of [CpFe(CO)_2_]I with the silanides Na[SiR*t*Bu_2_] (Lerner, 2005) in THF. The reaction of [CpFe(CO)_2_]*X* (*X* = halogen) with silyl thiolates [SSi*R*_3_]^-^ is a method to prepare the corresponding chalcogenolato complexes. We found that the sodium thiolate Na[SSi*t*Bu_3_] (Kückmann *et al.*, 2005, 2008, 2010) reacts readily with [CpFe(CO)_2_][BF_4_] in THF at room temperature to give the desired complex [CpFe(CO)_2_][SSi*t*Bu_3_] (Kückmann *et al.*, 2007). In this article we report the crystal structure of the starting material of this approach, [CpFe(CO)_2_][BF_4_]. The title compound [CpFe(CO)_2_][BF_4_] was easily accessible from the reaction of [CpFe(CO)_2_]I with AgBF_4_ in THF. Single crystals of the acetonitrile solvate [CpFe(CO)_2_(NCCH_3_)][BF_4_] were obtained by recrystallization from acetonitrile.

The crystal structure of the title compound (Fig. 1), of which only the coordinates of the non-H atoms of the cation have previously been determined (Fadel *et al.*, 1979) has been reinvestigated, now with the location of the tetrafluorido borate anion and improved *R* factors for the present model.

The Fe^II^ atom in the cation is coordinated by a cyclopentadienyl ring, two carbonyl ligands and an acetonitrile molecule displaying a three-legged piano-stool structure. Three of the four F atoms of the BF_4_^-^ anion are disordered over two sites with a site occupancy factor of 0.709 (10) for the major occupied site.

It is noteworthy, that Callan *et al.* (1987) determined the closely related structure of dicarbonyl-(η^5^-cyclopentadienyl)-(*N*-methyl cyanido)-iron(II) tetrafluoridoborate which has a methylisocyanide molecule bonded to the iron(II) atom instead of an acetonitrile molecule as in the title compound.

### Experimental

AgBF_4_ (0.19 g, 0.98 mmol) was added to a solution of [CpFe(CO)_2_]I (0.31 g, 1.02 mmol) (Fig. 1). The mixture was stirred at room temperature for 12 h. After filtration the solvent was removed *in vacuo*. Single crystals of the acetonitrile solvate [CpFe(CO)_2_(NCCH_3_)][BF_4_] were obtained by recrystallization from acetonitrile (yield: 74 mol%).

### Refinement

H atoms bonded to C were refined using a riding model, with C—H = 0.95 Å and with *U*_iso_(H) = 1.2*U*_eq_(C) or with C—H = 0.98 Å and with *U*_iso_(H) = 1.5*U*_eq_(C_methyl_). The methyl group was allowed to rotate but not to tip. Three of the F atoms of the BF_4_^-^ anion are disordered over two sites with a site occupancy factor of 0.709 (10) for the major occupied site and were refined with isotropic displacement parameters. The B—F bond lengths and the F···F distances were restrained to be equal.

### Crystal data

**Table:**

[Fe(C_5_H_5_)(C_2_H_3_N)(CO)_2_]BF_4_	*F*(000) = 608
*M**_r_* = 304.82	*D*_x_ = 1.689 Mg m^−^^3^
Monoclinic, *P*2_1_/*c*	Mo *K*α radiation, λ = 0.71073 Å
Hall symbol: -P 2ybc	Cell parameters from 6543 reflections
*a* = 6.8842 (7) Å	θ = 3.6–25.7°
*b* = 15.289 (2) Å	µ = 1.30 mm^−^^1^
*c* = 11.4353 (12) Å	*T* = 173 K
β = 95.192 (8)°	Rod, orange
*V* = 1198.7 (2) Å^3^	0.33 × 0.12 × 0.12 mm
*Z* = 4	

### Data collection

**Table:**

Stoe IPDS II two-circle diffractometer	2222 independent reflections
Radiation source: fine-focus sealed tube	1976 reflections with *I* > 2σ(*I*)
Graphite monochromator	*R*_int_ = 0.044
ω scans	θ_max_ = 25.6°, θ_min_ = 3.6°
Absorption correction: multi-scan (*MULABS*; Spek, 2009; Blessing, 1995)	*h* = −8→8
*T*_min_ = 0.674, *T*_max_ = 0.860	*k* = −18→15
6372 measured reflections	*l* = −13→13

### Refinement

**Table:**

Refinement on *F*^2^	Primary atom site location: structure-invariant direct methods
Least-squares matrix: full	Secondary atom site location: difference Fourier map
*R*[*F*^2^ > 2σ(*F*^2^)] = 0.055	Hydrogen site location: inferred from neighbouring sites
*wR*(*F*^2^) = 0.151	H-atom parameters constrained
*S* = 1.04	*w* = 1/[σ^2^(*F*_o_^2^) + (0.0822*P*)^2^ + 3.3996*P*] where *P* = (*F*_o_^2^ + 2*F*_c_^2^)/3
2222 reflections	(Δ/σ)_max_ < 0.001
162 parameters	Δρ_max_ = 1.43 e Å^−^^3^
45 restraints	Δρ_min_ = −0.87 e Å^−^^3^

### Special details

**Table:**

Geometry. All e.s.d.'s (except the e.s.d. in the dihedral angle between two l.s. planes) are estimated using the full covariance matrix. The cell e.s.d.'s are taken into account individually in the estimation of e.s.d.'s in distances, angles and torsion angles; correlations between e.s.d.'s in cell parameters are only used when they are defined by crystal symmetry. An approximate (isotropic) treatment of cell e.s.d.'s is used for estimating e.s.d.'s involving l.s. planes.
Refinement. Refinement of *F*^2^ against ALL reflections. The weighted *R*-factor *wR* and goodness of fit *S* are based on *F*^2^, conventional *R*-factors *R* are based on *F*, with *F* set to zero for negative *F*^2^. The threshold expression of *F*^2^ > σ(*F*^2^) is used only for calculating *R*-factors(gt) *etc*. and is not relevant to the choice of reflections for refinement. *R*-factors based on *F*^2^ are statistically about twice as large as those based on *F*, and *R*- factors based on ALL data will be even larger.

### Fractional atomic coordinates and isotropic or equivalent isotropic displacement parameters (Å^2^)

**Table:**

	*x*	*y*	*z*	*U*_iso_*/*U*_eq_	Occ. (<1)
B1	0.1682 (8)	0.4317 (5)	0.8015 (5)	0.0504 (17)	
F1	0.0863 (6)	0.3739 (3)	0.8730 (4)	0.0696 (11)	
F2	0.0456 (9)	0.4609 (4)	0.7113 (5)	0.073 (2)*	0.709 (10)
F3	0.1922 (8)	0.5136 (3)	0.8728 (5)	0.0584 (17)*	0.709 (10)
F4	0.3529 (8)	0.4133 (4)	0.7734 (7)	0.0631 (18)*	0.709 (10)
F2'	0.126 (3)	0.3804 (14)	0.6880 (14)	0.122 (8)*	0.291 (10)
F3'	0.104 (3)	0.5108 (11)	0.773 (2)	0.127 (9)*	0.291 (10)
F4'	0.3687 (15)	0.4177 (9)	0.8167 (15)	0.056 (4)*	0.291 (10)
Fe1	0.70458 (8)	0.33865 (4)	0.44357 (5)	0.0232 (3)	
C1	0.9179 (7)	0.3351 (3)	0.3623 (4)	0.0284 (9)	
O1	1.0553 (5)	0.3298 (2)	0.3141 (4)	0.0424 (9)	
C2	0.7445 (6)	0.4506 (3)	0.4916 (4)	0.0309 (10)	
O2	0.7644 (6)	0.5205 (3)	0.5253 (4)	0.0477 (10)	
N1	0.5355 (5)	0.3759 (2)	0.3084 (3)	0.0260 (8)	
C3	0.4383 (6)	0.4036 (3)	0.2304 (4)	0.0268 (9)	
C4	0.3128 (7)	0.4408 (4)	0.1331 (4)	0.0390 (11)	
H4A	0.1890	0.4593	0.1613	0.058*	
H4B	0.2877	0.3966	0.0715	0.058*	
H4C	0.3776	0.4914	0.1011	0.058*	
C11	0.6770 (13)	0.2026 (4)	0.4651 (6)	0.064 (2)	
H11	0.7006	0.1607	0.4066	0.076*	
C12	0.8106 (10)	0.2334 (5)	0.5468 (7)	0.068 (2)	
H12	0.9432	0.2155	0.5566	0.081*	
C13	0.7255 (14)	0.2963 (5)	0.6162 (5)	0.075 (2)	
H13	0.7877	0.3293	0.6793	0.090*	
C14	0.5213 (12)	0.2996 (4)	0.5701 (7)	0.071 (2)	
H14	0.4216	0.3352	0.5973	0.085*	
C15	0.5027 (10)	0.2403 (5)	0.4787 (6)	0.0620 (19)	
H15	0.3846	0.2278	0.4321	0.074*	

### Atomic displacement parameters (Å^2^)

**Table:**

	*U*^11^	*U*^22^	*U*^33^	*U*^12^	*U*^13^	*U*^23^
B1	0.034 (3)	0.071 (5)	0.046 (3)	0.005 (3)	0.001 (3)	0.031 (3)
F1	0.083 (3)	0.062 (2)	0.066 (2)	−0.025 (2)	0.020 (2)	0.008 (2)
Fe1	0.0228 (4)	0.0248 (4)	0.0217 (4)	0.0011 (2)	0.0004 (2)	0.0004 (2)
C1	0.027 (2)	0.024 (2)	0.033 (2)	−0.0026 (16)	0.0002 (19)	−0.0044 (17)
O1	0.0292 (18)	0.043 (2)	0.057 (2)	−0.0040 (14)	0.0131 (17)	−0.0086 (17)
C2	0.025 (2)	0.036 (3)	0.031 (2)	0.0032 (18)	−0.0018 (17)	−0.006 (2)
O2	0.046 (2)	0.039 (2)	0.056 (2)	0.0030 (17)	−0.0039 (17)	−0.0191 (18)
N1	0.0210 (16)	0.0306 (19)	0.0264 (18)	−0.0055 (15)	0.0028 (15)	−0.0026 (15)
C3	0.0224 (19)	0.033 (2)	0.025 (2)	−0.0022 (17)	0.0016 (17)	−0.0002 (18)
C4	0.036 (2)	0.048 (3)	0.031 (2)	0.005 (2)	−0.006 (2)	0.004 (2)
C11	0.115 (6)	0.030 (3)	0.049 (4)	−0.005 (3)	0.028 (4)	0.006 (3)
C12	0.052 (4)	0.074 (5)	0.080 (5)	0.023 (3)	0.016 (4)	0.049 (4)
C13	0.132 (7)	0.065 (4)	0.024 (3)	−0.034 (5)	−0.012 (3)	0.017 (3)
C14	0.091 (5)	0.049 (4)	0.084 (5)	0.027 (4)	0.065 (5)	0.034 (4)
C15	0.060 (4)	0.062 (4)	0.061 (4)	−0.029 (3)	−0.008 (3)	0.033 (3)

### Geometric parameters (Å, º)

**Table:**

B1—F3'	1.319 (14)	C2—O2	1.140 (6)
B1—F2	1.348 (8)	N1—C3	1.147 (6)
B1—F1	1.361 (7)	C3—C4	1.461 (6)
B1—F4	1.369 (8)	C4—H4A	0.9800
B1—F4'	1.392 (11)	C4—H4B	0.9800
B1—F3	1.496 (9)	C4—H4C	0.9800
B1—F2'	1.521 (14)	C11—C12	1.336 (11)
Fe1—C1	1.809 (5)	C11—C15	1.353 (11)
Fe1—C2	1.811 (5)	C11—H11	0.9500
Fe1—N1	1.935 (4)	C12—C13	1.407 (11)
Fe1—C13	2.070 (6)	C12—H12	0.9500
Fe1—C12	2.088 (6)	C13—C14	1.457 (12)
Fe1—C14	2.091 (5)	C13—H13	0.9500
Fe1—C11	2.106 (6)	C14—C15	1.381 (11)
Fe1—C15	2.110 (6)	C14—H14	0.9500
C1—O1	1.140 (6)	C15—H15	0.9500
			
F3'—B1—F1	126.5 (11)	O1—C1—Fe1	177.0 (4)
F2—B1—F1	114.2 (5)	O2—C2—Fe1	177.2 (4)
F2—B1—F4	114.8 (6)	C3—N1—Fe1	175.4 (4)
F1—B1—F4	116.9 (6)	N1—C3—C4	178.5 (5)
F3'—B1—F4'	118.7 (12)	C3—C4—H4A	109.5
F1—B1—F4'	106.6 (8)	C3—C4—H4B	109.5
F2—B1—F3	99.8 (5)	H4A—C4—H4B	109.5
F1—B1—F3	104.3 (5)	C3—C4—H4C	109.5
F4—B1—F3	103.9 (6)	H4A—C4—H4C	109.5
F3'—B1—F2'	103.2 (13)	H4B—C4—H4C	109.5
F1—B1—F2'	96.9 (10)	C12—C11—C15	109.3 (6)
F4'—B1—F2'	98.0 (11)	C12—C11—Fe1	70.7 (4)
C1—Fe1—C2	94.4 (2)	C15—C11—Fe1	71.5 (4)
C1—Fe1—N1	93.21 (18)	C12—C11—H11	125.4
C2—Fe1—N1	91.61 (19)	C15—C11—H11	125.4
C1—Fe1—C13	119.4 (3)	Fe1—C11—H11	124.1
C2—Fe1—C13	90.6 (3)	C11—C12—C13	109.9 (6)
N1—Fe1—C13	147.0 (3)	C11—C12—Fe1	72.1 (4)
C1—Fe1—C12	90.5 (2)	C13—C12—Fe1	69.5 (4)
C2—Fe1—C12	121.4 (3)	C11—C12—H12	125.1
N1—Fe1—C12	146.4 (3)	C13—C12—H12	125.1
C13—Fe1—C12	39.6 (3)	Fe1—C12—H12	124.8
C1—Fe1—C14	156.4 (2)	C12—C13—C14	104.9 (6)
C2—Fe1—C14	98.3 (3)	C12—C13—Fe1	70.9 (3)
N1—Fe1—C14	106.2 (3)	C14—C13—Fe1	70.3 (3)
C13—Fe1—C14	41.0 (3)	C12—C13—H13	127.6
C12—Fe1—C14	65.9 (3)	C14—C13—H13	127.6
C1—Fe1—C11	96.7 (2)	Fe1—C13—H13	123.1
C2—Fe1—C11	155.7 (2)	C15—C14—C13	105.7 (6)
N1—Fe1—C11	109.3 (3)	C15—C14—Fe1	71.6 (3)
C13—Fe1—C11	65.1 (3)	C13—C14—Fe1	68.7 (3)
C12—Fe1—C11	37.2 (3)	C15—C14—H14	127.1
C14—Fe1—C11	64.6 (3)	C13—C14—H14	127.1
C1—Fe1—C15	131.0 (3)	Fe1—C14—H14	124.2
C2—Fe1—C15	134.4 (3)	C11—C15—C14	110.2 (7)
N1—Fe1—C15	90.2 (2)	C11—C15—Fe1	71.1 (4)
C13—Fe1—C15	65.5 (3)	C14—C15—Fe1	70.1 (4)
C12—Fe1—C15	63.0 (3)	C11—C15—H15	124.9
C14—Fe1—C15	38.4 (3)	C14—C15—H15	124.9
C11—Fe1—C15	37.4 (3)	Fe1—C15—H15	125.5
			
C1—Fe1—C11—C12	82.0 (4)	N1—Fe1—C13—C14	8.0 (7)
C2—Fe1—C11—C12	−34.5 (9)	C12—Fe1—C13—C14	−114.4 (6)
N1—Fe1—C11—C12	177.7 (4)	C11—Fe1—C13—C14	−79.2 (5)
C13—Fe1—C11—C12	−37.4 (5)	C15—Fe1—C13—C14	−37.9 (4)
C14—Fe1—C11—C12	−82.9 (5)	C12—C13—C14—C15	−0.4 (6)
C15—Fe1—C11—C12	−119.1 (6)	Fe1—C13—C14—C15	62.5 (4)
C1—Fe1—C11—C15	−158.9 (4)	C12—C13—C14—Fe1	−62.9 (4)
C2—Fe1—C11—C15	84.6 (8)	C1—Fe1—C14—C15	−75.5 (10)
N1—Fe1—C11—C15	−63.1 (4)	C2—Fe1—C14—C15	162.8 (4)
C13—Fe1—C11—C15	81.7 (5)	N1—Fe1—C14—C15	68.7 (4)
C12—Fe1—C11—C15	119.1 (6)	C13—Fe1—C14—C15	−115.8 (6)
C14—Fe1—C11—C15	36.2 (5)	C12—Fe1—C14—C15	−76.4 (5)
C15—C11—C12—C13	−1.8 (7)	C11—Fe1—C14—C15	−35.3 (4)
Fe1—C11—C12—C13	59.5 (5)	C1—Fe1—C14—C13	40.4 (10)
C15—C11—C12—Fe1	−61.4 (4)	C2—Fe1—C14—C13	−81.3 (4)
C1—Fe1—C12—C11	−100.4 (4)	N1—Fe1—C14—C13	−175.4 (4)
C2—Fe1—C12—C11	164.1 (4)	C12—Fe1—C14—C13	39.5 (5)
N1—Fe1—C12—C11	−3.8 (7)	C11—Fe1—C14—C13	80.5 (5)
C13—Fe1—C12—C11	120.1 (6)	C15—Fe1—C14—C13	115.8 (6)
C14—Fe1—C12—C11	79.2 (5)	C12—C11—C15—C14	1.6 (7)
C15—Fe1—C12—C11	36.6 (4)	Fe1—C11—C15—C14	−59.3 (4)
C1—Fe1—C12—C13	139.5 (5)	C12—C11—C15—Fe1	60.9 (5)
C2—Fe1—C12—C13	44.0 (5)	C13—C14—C15—C11	−0.6 (7)
N1—Fe1—C12—C13	−124.0 (5)	Fe1—C14—C15—C11	60.0 (4)
C14—Fe1—C12—C13	−40.9 (5)	C13—C14—C15—Fe1	−60.6 (4)
C11—Fe1—C12—C13	−120.1 (6)	C1—Fe1—C15—C11	28.2 (5)
C15—Fe1—C12—C13	−83.5 (5)	C2—Fe1—C15—C11	−145.0 (4)
C11—C12—C13—C14	1.4 (7)	N1—Fe1—C15—C11	122.6 (4)
Fe1—C12—C13—C14	62.5 (4)	C13—Fe1—C15—C11	−80.4 (5)
C11—C12—C13—Fe1	−61.1 (5)	C12—Fe1—C15—C11	−36.3 (4)
C1—Fe1—C13—C12	−48.2 (5)	C14—Fe1—C15—C11	−120.8 (6)
C2—Fe1—C13—C12	−143.6 (5)	C1—Fe1—C15—C14	149.1 (4)
N1—Fe1—C13—C12	122.5 (6)	C2—Fe1—C15—C14	−24.1 (6)
C14—Fe1—C13—C12	114.4 (6)	N1—Fe1—C15—C14	−116.6 (4)
C11—Fe1—C13—C12	35.2 (5)	C13—Fe1—C15—C14	40.4 (5)
C15—Fe1—C13—C12	76.5 (5)	C12—Fe1—C15—C14	84.5 (5)
C1—Fe1—C13—C14	−162.7 (4)	C11—Fe1—C15—C14	120.8 (6)
C2—Fe1—C13—C14	102.0 (4)		
